# Supplementary figures and images for: Development of a Method for Scaffold-Free Elastic Cartilage Creation
Source: Int J Mol Sci. 2020 Nov 11;21(22):8496. doi: 10.3390/ijms21228496 (PMC7698291; doi:10.3390/ijms21228496)

A

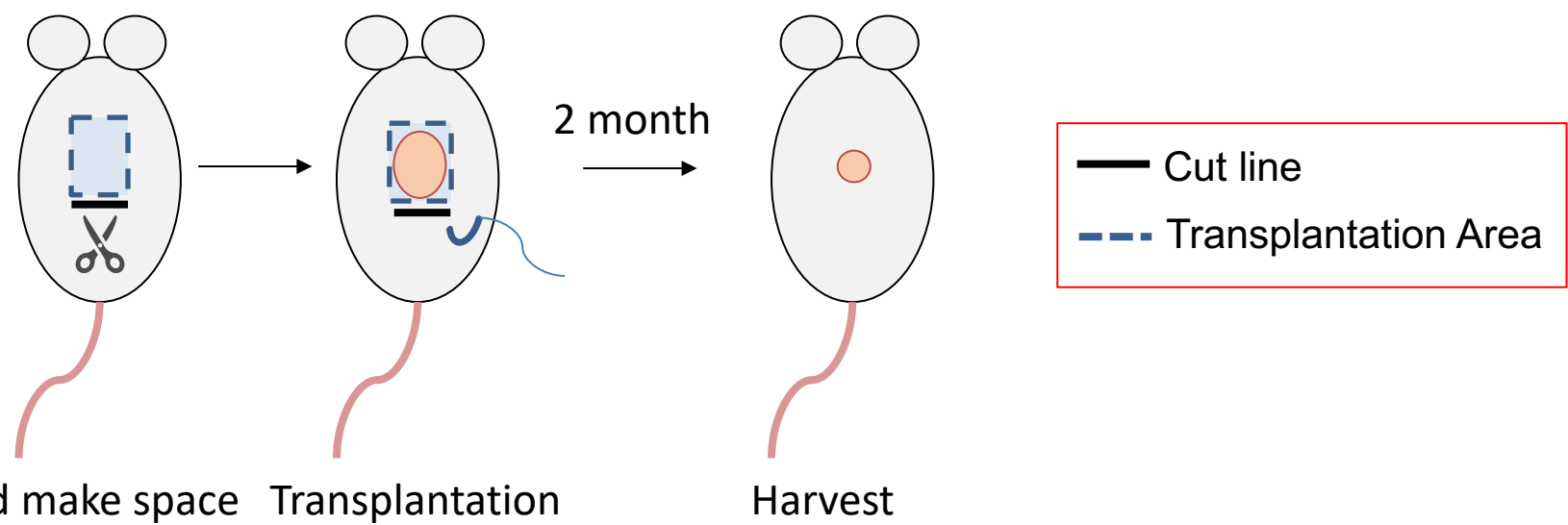

B

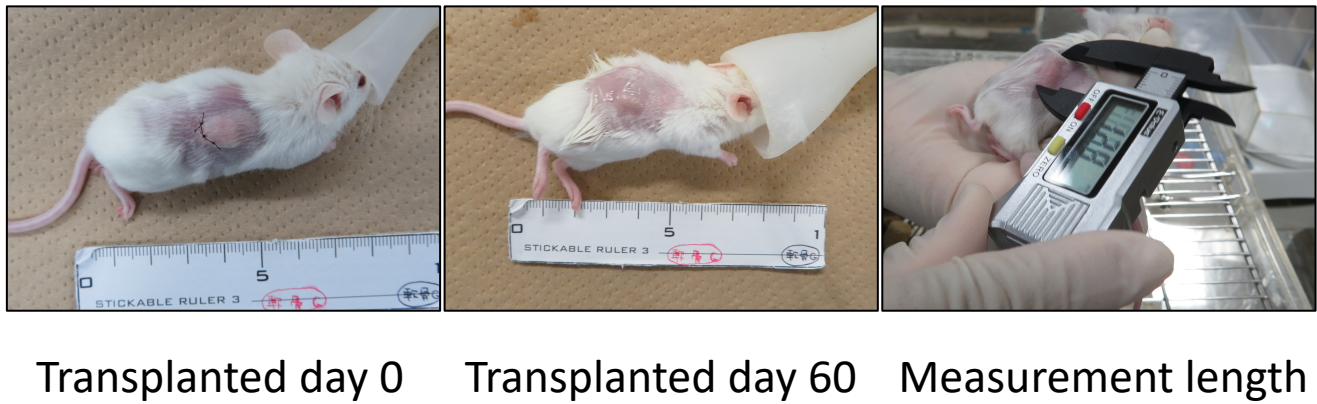

## Sham

HE

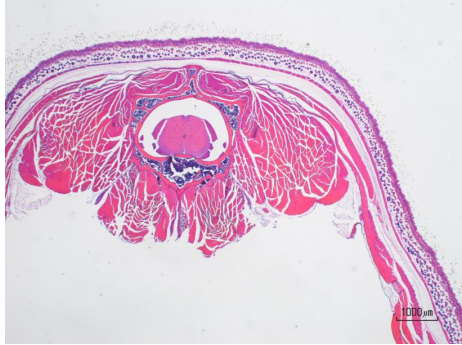

AB

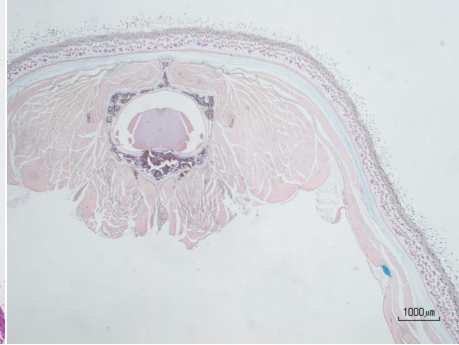

## Transplantation

HE

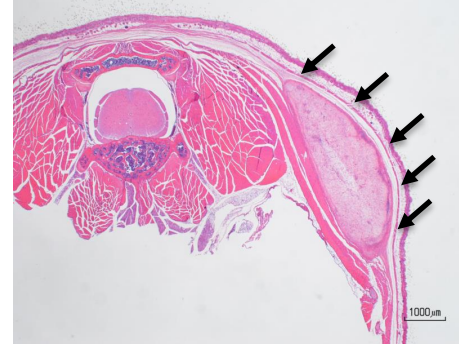

AB

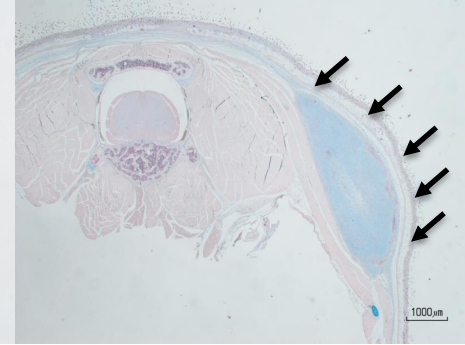

Supplement: Supplementary file 1 [file ijms-21-08496-s001.pdf]
